# The Home Literacy Environment as a Mediator Between Parental Attitudes Toward Shared Reading and Children’s Linguistic Competencies

**DOI:** 10.3389/fpsyg.2020.01628

**Published:** 2020-07-21

**Authors:** Frank Niklas, Astrid Wirth, Sabrina Guffler, Nadja Drescher, Simone C. Ehmig

**Affiliations:** ^1^Department of Psychology, Ludwig-Maximilians-University, Munich, Germany; ^2^Department of Educational Psychology, Julius-Maximilians-University, Würzburg, Germany; ^3^German Reading Foundation, Mainz, Germany

**Keywords:** home literacy environment, parental attitude toward reading, linguistic competencies, kindergarten children, development of early child competencies

## Abstract

The home learning environment plays an important role for children’s early competencies development. In particular, the early home literacy environment (HLE) that consists of all literacy resources and interactions in a family that support children’s linguistic and literacy learning is closely associated with children’s language comprehension and production. A key aspect of the HLE is shared reading that should start early in children’s life and should be part of a regular routine in the family. However, parental attitudes toward (shared) reading have hardly been analyzed.

In this longitudinal study, we analyzed the associations between parental attitudes toward shared reading and children’s linguistic competencies and whether these associations may be mediated by the HLE. Further, we were interested in changes of parental attitudes over time and their association with child and family background characteristics. The sample consisted of *N* = 133 children with an average age of about 3 years at t1. Children were tested two more times with a 6-month period in-between each assessment. Parental attitudes toward shared reading, socioeconomic status (SES), and the HLE were assessed *via* parental survey. Children’s sentence comprehension, productive language, and grammar were measured with a standardized test battery. Children whose parents had a more positive attitude toward shared reading not only lived in a greater quality HLE but also performed better in the linguistic tests. In a structural equation model, an indirect effect was found showing that the HLE mediated the effect of parental attitudes on children’s linguistic competencies. Further, parental attitudes toward shared reading did not change significantly across t1 to t3, and a lower score in the SES scale was associated with a less positive attitude toward shared reading. Consequently, parental attitudes toward shared reading seem to be an important basis for individual differences in the quality of the HLE and also for children’s linguistic competencies. As these attitudes vary in the context of different family SES backgrounds, they may be a good target for interventions to support the quality of the HLE and young children’s linguistic learning.

## Introduction

Attitudes are of great interest for psychologists and educators as attitudes influence our perception and may have an impact on our behavior (cf. Eysenck, 2004; Schwarz, 2007). In the family context, parental attitudes play a major role for young children as parents are very attractive role models for their children (cf. Niklas, 2015). Further, parents create the environment their children experience, and thus parental attitudes are most likely to influence the home learning environment and children’s learning within this context (e.g., Bingham, 2007; Park, 2008; Skibbe et al., 2008).

Shared reading with children is a key aspect of the home literacy environment (HLE) that supports children’s development of linguistic and literacy competencies (Niklas et al., 2016b). However, although shared reading is deemed important by most parents in Germany, some children, and in particular, children from families with a low socioeconomic status (SES), are rarely read to (German Reading Foundation, 2010). As maternal literacy beliefs are closely associated with the HLE and child outcomes (Weigel et al., 2006), such attitudes may be a good target for interventions.

In this study, we analyze the associations of parental attitudes toward shared reading, the quality of the HLE, and young children’s linguistic outcomes in a longitudinal design. Further, we were interested in whether parental attitudes change across 1 year and whether these attitudes were associated with child and family characteristics.

### The Development of Children’s Early Linguistic Competencies

An important early linguistic ability is the ability to understand spoken language, often referred to as language comprehension skills. Language comprehension skills consist of basic abilities such as the activation of word meanings and understanding sentences, of receptive vocabulary, the knowledge of text and sentence structures, and language production skills such as children’s expressive vocabulary (Lepola et al., 2016; Niklas et al., 2016a). Both receptive and expressive language skills are closely related (e.g., Cutting and Dunn, 1999). Further, these abilities are highly stable competencies from kindergarten age onward (Whitehurst and Lonigan, 2001).

In their concept of emergent literacy, Whitehurst and Lonigan (1998) differentiate between such language competencies as outside-in skills and inside-out skills such as phonological awareness and letter knowledge. Indeed, inside-out skills are also important predictors of later reading and writing abilities; however, these skills develop at a later age and are not formally taught in German kindergartens and preschools (Niklas and Schneider, 2015). As our analytic sample consists of 3- to 4-year-old children, we only focus on outside-in skills.

Early linguistic and literacy competencies are essential for a successful school career, and precursors of these abilities develop long before children enter school. An early assessment of these skills is preferable, as specific precursors of later literacy competencies such as language comprehension and production are important predictors of academic performance in school (e.g., Joshi, 2005; Juel, 2006; Claessens et al., 2009). Consequently, precursors of literacy abilities and children’s later literacy competencies lie on a continuum (e.g., Torppa et al., 2007; Lepola et al., 2016). Further, individual differences in vocabulary and language comprehension skills in early years predict not only later reading abilities but also motivational and behavioral outcomes in children (Laitinen et al., 2017).

### The Home Literacy Environment and Early Linguistic Competencies

Children develop early linguistic competencies during the interaction with their parents (Vygotsky, 1978). Consequently, the HLE provides numerous opportunities for teaching and learning activities that support the development of children’s linguistic and literacy abilities (Niklas and Schneider, 2017a). The HLE is a multifaceted construct comprising current parental reading habits, shared reading habits in the family, and more general aspects of family literacy such as the frequency of library visits and the number of books in a household. These aspects can be further differentiated into a cultural capital and a cultural praxis (e.g., Niklas et al., 2013). Whereas in the context of the HLE cultural capital refers to the number of books and children’s books in a household, cultural praxis consists of all literacy activities in the family such as shared reading. Both aspects are closely associated; however, they may still differ in the role they play for the development of children’s linguistic competencies (e.g., McElvany et al., 2009).

The association between the HLE and children’s linguistic and literacy competencies is also evident in intervention studies that try to enhance the quality of the HLE to support children’s competency development. Indeed, various family literacy programs have demonstrated small to large effects (e.g., Harper et al., 2011; Lever and Sénéchal, 2011). For instance, Niklas and Schneider (2015, 2017b) showed that even non-intensive interventions that just comprised one parent evening and one individual session may change the HLE and subsequently impact on children’s development of their vocabulary and phonological awareness.

The observation that the HLE and subsequent child competencies can be improved by interventions has been also confirmed in comprehensive meta-analyses. Sénéchal and Young (2008) and Mol et al. (2008) each analyzed 16 intervention studies that focused either on parental involvement in kindergarten and primary school children’s development of reading and spelling abilities or on dialogic reading (for more information on dialogic reading, see Cohrssen et al., 2016) and its effect on children’s vocabulary. Mean effect sizes of Cohen’s *d* = 0.65 and 0.42, and thus small to medium effects were found. Consequently, the HLE is a very important factor in children’s development of linguistic and literacy competencies.

### Parental Attitudes Toward Shared Reading

Some studies explicitly regard parental attitudes toward literacy as an aspect of a broader construct of the HLE (e.g., Niklas and Schneider, 2017b) or differentiate between the HLE and these attitudes as separate variables (e.g., Park, 2008), whereas in other studies on the HLE, attitudes are not taken into account (e.g., Niklas and Schneider, 2013). As children learn by interacting with and observing more knowledgeable others, in the early years often their parents (Vygotsky, 1978), they also take notice of parental attitudes displayed during these interactions and observations. Parents act as important role models for their young children (Bandura, 1977), and their attitudes are very likely to impact on children’s own attitudes and interests. Consequently, it is to be expected that parental attitudes toward shared reading shape children’s interest in literacy and books and in turn may also impact on children’s linguistic and literacy competencies (Bingham, 2007; Skibbe et al., 2008). Therefore, parental attitudes toward reading and literacy in general, and in families with young children, the attitudes toward shared reading, specifically, may be important for children’s development (Weigel et al., 2006; cf. Niklas, 2015).

According to the model of Zanna and Rempel (1988), objects are evaluated according to three different components: (1) cognitive, (2) affective, and (3) behavioral. In regard to shared reading, this model implies that parents will put a certain value on shared reading, will feel more or less positive about it, and finally, initiate shared reading session more or less often with their children and in a way that triggers more or less reading motivation. The attitude toward shared reading develops over time, may change from situation to situation, and will be closer associated with actual behavior when specific and concrete attitudes are assessed (cf. Schwarz and Bohner, 2001). However, given that attitudes also comprise a behavioral component, it is likely that parental attitudes toward shared reading will be closely associated with the HLE, in particular with the cultural practice (cf. Niklas et al., 2013). Actually, in a study by Tambyraja et al. (2017), caregivers’ own reading habits were a predictor of the general HLE in the family.

The development of parental attitudes toward shared reading depends on various experiences the parents had encountered such as their own shared reading experiences as children and in general their socialization (cf. Eysenck, 2004). Consequently, it is to be expected that the attitude toward shared reading should be associated with the socioeconomic status (SES) of the family (e.g., Park, 2008; Skibbe et al., 2008; Becker and McElvany, 2018), similar to the association of the SES with the HLE (Aikens and Barbarin, 2008; Niklas et al., 2013). When trying to tackle different linguistic and literacy outcomes of children from different family backgrounds, parental attitudes might be a worthwhile target.

### Research Focus

The association between the HLE and children’s early and later linguistic and literacy outcomes is well established (e.g., Sénéchal and LeFevre, 2002; Niklas and Schneider, 2013; Hemmerechts et al., 2017). However, less is known about the role parental attitudes toward shared reading play in this association, in particular for younger children (for an example, see Bingham, 2007). Further, it is still not clear whether we see changes in these attitudes across time and whether they are associated with child and family characteristics as many studies in this context only used cross-sectional data (e.g., Park, 2008; Hemmerechts et al., 2017).

We analyzed the development of child competencies across a 1-year period and assessed parental attitudes toward shared reading, the HLE, and linguistic outcomes. Here, we expected the parental attitudes toward reading and the quality of the HLE to be stable across the 1-year period (cf. Niklas, 2015). Further, we expected that a more positive attitude toward shared reading and a greater quality in the HLE should be associated with greater linguistic competencies in children. Finally, we assumed that the HLE should act as a mediator between parental attitudes and child outcomes.

## Materials and Methods

### Sample

In total, *N* = 133 children were assessed using a longitudinal research design with three measurement points (t1–t3) across 12 months (6 months in-between each measurement). Power analysis with G^∗^Power (Faul et al., 2007) indicated a sample size above *N* = 129 participants to be sufficient for the planned analyses. At t1, children were between 26 and 45 months old (*M* = 36.6, SD = 4.1). In the sample, gender was almost equally distributed, with 46% girls (*N* = 61). More than a third of the children (37.6%, *N* = 50) had a migration background with at least one parent being born outside of Germany.

All participating parents were asked about their occupation and their partner’s occupation to assign prestige values to these occupations (Wegener, 1988; cf. Christoph, 2005). Here, values ranged from 20 (an unskilled laborer) to 186.8 (a physician), and for the analyses, the highest prestige score in the household was used. Information about the SES could be obtained from *N* = 122 families with a mean of *M* = 86.86 (SD = 40.53), a value assigned to the occupation of a salesman.

### Procedure

Formal consent to conduct the study was obtained from the center coordinators and parents, and ethics approval was obtained from the University of Würzburg, Germany. Randomly selected kindergartens in two German states were contacted and invited to participate in our study. In Germany, most children are enrolled in kindergarten from 2 to 3 years of age until the beginning of formal schooling at the age of 6. Kindergarten refers to a nursery school or preschool setting, with a focus on playing and practical activities (see further Niklas et al., 2018). *N* = 21 kindergartens agreed to participate and handed out information and consent forms for all parents with children in the age group between 26 and 45 months. In each participating kindergarten, between *N* = 4 and *N* = 13 children (and their parents) participated in our study. At each measurement point, trained psychologists assessed children’s competencies in their kindergartens, whereas parents were asked to fill in surveys. Parental response rates lay between 84.1% and 75.8% for each measurement point (between *N* = 21 and *N* = 32 parents did not return the survey). Between *N* = 12 and *N* = 21 of the study children could not be tested at least once due to absence or refusal to participate. We address the handling of missing data in our analytic approach.

### Surveys and Test Instruments

At each measurement point, parents were asked about their family’s HLE and their attitude toward shared reading. Further, they were asked to provide information about their family background.

#### Home Literacy Environment

The HLE survey was an adapted version of a survey used by Niklas et al. (2016a). This survey contained seven items covering different facets of the HLE: the number of books at home, the number of children’s books at home, the frequency of reading to the child and the frequency of both parents’ own reading, the frequency of the child looking at picture books, and the frequency of library visits with the child. Each item had a range from 0 to 4. Both items concerning the number of (children’s) books at home were used to estimate the cultural literacy capital in a family (example: “How many children’s books does your child own?”), whereas the remaining five items assessed a family’s cultural literacy praxis (example: “How often do you read to your child?”; cf. Niklas et al., 2013). The cultural capital scale had a maximum attainable sum score of 8, with Cronbach’s α = 0.78 at t1, 0.86 at t2, and 0.84 at t3. The cultural praxis scale had a maximum attainable sum score of 20, with Cronbach’s α = 0.67 at t1, 0.60 at t2, and 0.65 at t3. The sum score of the global HLE scale was a reliable measure (Cronbach’s α = 0.78 at t1, 0.74 at t2, and 0.71 at t3) with a maximum attainable score of 28. Retest reliability for the global HLE scale was high, with *r*_12_ = 0.80, *r*_13_ = 0.71, and *r*_23_ = 0.79.

#### Parental Attitudes

In the parent survey, 11 items assessed attitudes, behaviors, and family situations in the context of shared reading. We conducted an exploratory factor analysis in SPSS to identify common factors. Only the first factor explained a major part of the variance, and four items on cognitive attitudes loaded on this factor. All other items either did not load on a specific factor or were the only items to load on an additional factor. Consequently, we assessed parental attitudes toward shared reading with four items on 5-point Likert scales. The items ranged from 0 (I do not agree) to 4 (I agree completely). Here, all items focused on cognitive attitudes toward shared reading and assessed the value attached to reading at home, perceived interest in reading by the child, and parental motivation toward reading and shared reading (item example: “Reading is regarded as an important activity at our home”). The parental attitudes toward shared reading subscale had a maximum attainable sum score of 16, with *Cronbach’s* α = 0.90 at t1, 0.90 at t2, and 0.86 at t3. Parental attitudes toward shared reading were fairly stable across t1 to t3, with *r*_12_ = 0.60, *r*_13_ = 0.73, and *r*_23_ = 0.71. The four items assessing cognitive attitudes toward shared reading had been used in previous studies (e.g., Park, 2008; Niklas et al., 2016a; Wirth et al., 2019).

#### Linguistic Abilities

Children’s level of linguistic abilities was assessed with the standardized German language development test instrument SETK 3-5 (Grimm et al., 2010) that comprises subscales for language comprehension and language production. Reliability (Cronbach’s α) was at least α = 0.70 for each subscale (Neugebauer and Becker-Mrotzek, 2013). Each subtest started with a sample item to demonstrate how to approach the question and to provide feedback for the child. During the test phase, no further feedback was given.

At t1 and t2, the language comprehension scale consisted of three subtests. In the first one, children were asked to select a picture out of four similar pictures, matching the sentence that had been read out to them (nine items, maximum attainable score of 9). In the following two subtests, children were asked to act according to short instructions (for example, “Put the red buttons on the box”). Both subtests consisted of five items each, with a maximum attainable score of 5, respectively. At t3, subtest 1 was omitted due to children’s age and according to the test manual and instead another five items were added in which children were asked to act according to more complex instructions (for example, “The yellow ball, that is pushed by the white ball, falls from the table”). Consequently, the attainable maximum score was lower at t3 compared to t1 and t2.

Language production consisted of two subtests for t1 and t2, assessing the encoding of semantic relations and morphological rule-making. Both subtests were z-transformed and summed up into the language production scale at t1 and t2. At t3, when all participating children were older than 3 years old, language production was assessed with a more comprehensive morphological rule-making test. To be consistent, this test was also z-transformed.

In the subtest “encoding of semantic relations,” children were asked to describe 11 pictures to assess their use of prepositions (for example, “The children walk across the street.”). There is no maximum attainable score as children were free to describe pictures with an unlimited number of words, which were counted for each child individually. In the subtest “morphological rule-making,” children were asked to say plural forms of different nouns (for example, “car–cars”). Here, the maximum attainable score was 20 at t1 and t2 and 36 at t3 due to eight additional test items.

We created an index score combining both z-transformed language comprehension and language production scales into a general linguistic abilities scale. Retest reliability for the general linguistic abilities was very high, with *r*_12_ = 0.85, *r*_13_ = 0.75, and *r*_23_ = 0.88.

#### Non-verbal Intelligence

In addition to children’s age, sex, and their family’s SES, all analyses were controlled for children’s non-verbal intelligence. Children’s non-verbal intelligence was assessed at t1 and t3 with the Columbia Mental Maturity Scale (CMM; Burgemeister et al., 1972), assessing 3- to 5-year-old children’s capability for abstraction and logical reasoning. Here, children had to identify the odd picture in an array of three to five pictures (e.g., four identical dogs and one cat), and a maximum of 57 points was attainable. Split-half reliability in German contexts ranges from 0.92 to 0.96, and the CMM proved to be a good indicator of children’s general cognitive abilities in recent German studies (Esser, 2002; Hasselhorn et al., 2012; Niklas and Schneider, 2017a).

All descriptive data and the sample sizes for all variables are shown in Table 1.

**TABLE 1:** Descriptive statistics for the study variables at t1, t2, and t3 (sample sizes, means, standard deviations, observed ranges).

Variables	*N*	*M*	SD	Observed range
Intelligence	121/-/112	27.8/-/38.2	13.4/-/10.7	0.0–50.0/–/0.0–54.0
SES^1^	122	86.89	40.84	20.00–186.80
HLE	108/100/94	19.2/19.5/19.4	4.5/4.2/4.2	5.0–26.0/6.0–26.0/7.0–26.0
HLE–Cultural capital	111/102/96	7.02/7.15/7.29	1.56/1.45/1.37	2.0–8.0/2.0–8.0/2.0–8.0
HLE–Cultural praxis	110/103/103	12.07/12.41/12.41	3.47/3.18/3.18	3.0–18.0/3.0–18.0/3.0–18.0
Parental attitudes	109/101/94	12.43/12.89/12.84	3.55/3.19/2.73	0.0–16.0/3.0–16.0/5.0–16.0
Linguistic abilities^2^	114/112/101	0.53/0.01/0.78	3.19/3.19/3.78	-5.5 to 7.8/-7.6 to 5.5/-8.9 to 7.3
Language comprehension^3^	118/116/112	7.94/10.99/8.00	4.67/5.06/4.28	0.0–19.0/0.0–18.0/0.0–15.0
Language production^4^	115/113/112	-0.01/0.01/0.00	1.77/1.85/1.00	-2.9 to 4.1/-3.7 to 2.9/-2.0–1.9

*^1^SES, highest family occupational prestige; ^2^combined index of the z-transformed language comprehension, and language production scale of the SETK; ^3^ the subtests changed between t2 and t3; ^4^z-transformed subscale with two subscales at t1 and t2 and one subtest at t3. HLE, home literacy environment; SES, socioeconomic status.*

### Analytic Approach

Data analyses were conducted using SPSS 24 (Ibm Corp, 2016) for descriptive and correlative analyses and Mplus 7 (Muthén and Muthén, 2012) for structural equation modeling (SEM). As some parental surveys were not or only partially completed, and some children’s test scores were missing, several cases were incomplete. After analyzing the missing data for patterns, we estimated missing data using the full information maximum likelihood option (MLR estimator) in Mplus.

First, results of bivariate correlational analyses (Pearson’s *r*) of all study variables for the three measurement points are presented. Here, we analyzed whether parental attitudes and HLE were associated with the control variables and the linguistic outcomes. In a second step, we carried out univariate variance analyses with repeated measurement (rm ANOVA) to test whether parental attitudes toward shared reading and the quality of the HLE varied across t1 to t3.

Finally, we used SEM to analyze the association between parental attitudes toward shared reading and children’s linguistic competencies and whether this association may be mediated by the HLE. We examined this association controlling for various child and family characteristics.

## Results

### Correlational Analyses

Table 2 shows the cross-sectional results for the correlational analyses at t1, t2, and t3.

**TABLE 2:** Cross-sectional correlational analyses for all study variables at t1, t2, and t3.

	2	3	4	5	6	7	8	9	10	11
Age (1)	**0.22**/-/0.19	-0.14/-0.13/-0.08	-0.08/-0.06/-0.04	-0.09**/**-0.10/-0.09	-0.02/-0.13**/**-**0.21**	-0.13/-0.08/-0.13	0.01/-0.05/-0.19	**0.40**/**0.29**/**0.22**	**0.33/0.28**/0.18	**0.37/0.29/**0.12
Intelligence^1^ (2)	–	0.00/-/-0.00	0.08/-/**0.30**	0.03/-/**0.26**	0.05/-/**0.21**	0.02/-/0.14	0.07/-/**0.23**	**0.45**/-/**0.24**	**0.37/-/0.46**	**0.36/-/0.47**
Sex^2^ (3)	–	–	-0.07	-0.09/-0.11/-0.10	-0.12/-0.11/-0.12	-0.05/-0.09/-0.09	-0.11/-0.06/-0.08	-**0.25/**-**0.24/**-0.17	-**0.20**/-0.18/-0.16	-**0.28/**-**0.21/**-0.15
SES^3^ (4)	–	–	–	**0.58/0.51/0.53**	**0.53/0.48**/**0.41**	**0.55/0.44/0.44**	**0.50/0.40/0.54**	**0.46/0.43/0.41**	**0.48/0.50/0.49**	**0.47/0.38/0.44**
HLE (5)	–	–	–	–	**0.80/0.78/0.64**	**0.96/0.96/0.75**	**0.69/0.59/0.57**	**0.37**/**0.46**/**0.49**	**0.41/0.48/0.55**	**0.40/0.44/0.55**
HLE–Cultural capital (6)	–	–	–	–	–	**0.60/0.57/0.38**	**0.61/0.62/0.49**	**0.44/0.42/0.40**	**0.43/0.41/0.42**	**0.45/0.46/0.52**
HLE–Cultural praxis (7)	–	–	–	–	–	–	**0.65/0.49/0.52**	**0.33/0.41/0.47**	**0.33/0.43/0.53**	**0.31/0.42/0.54**
Parental attitudes (8)	–	–	–	–	–	–	–	**0.37/0.39/0.39**	**0.38/0.41/0.44**	**0.31/0.40/0.48**
Linguistic abilities^4^ (9)	–	–	–	–	–	–	–	–	**0.89/0.88/0.79**	**0.92/0.91/0.82**
Language comprehension (10)	–	–	–	–	–	–	–	–	–	**0.75/0.79/0.74**
Language production (11)	–	–	–	–	–	–	–	–	–	–

*Pearson’s *r* correlation coefficients; *p* < 0.05 in bold characters, *N* = 78–133. HLE, home literacy environment; SES, socioeconomic status. ^1^Children’s non-verbal intelligence (CMM) was assessed at t1 and t3; ^2^female = 0, male = 1; ^3^highest family occupational prestige; ^4^combined index of the z-transformed language comprehension, and language production scales of the SETK.*

As expected, significant medium effect size correlations were observed between the HLE with its subscales cultural praxis and cultural capital and children’s linguistic abilities at all three measurement points (*r* = 0.37–0.55) as well as large effect size correlations of the HLE with parental attitudes toward shared reading (*r* = 0.49–0.65). Further, linguistic abilities were also significantly correlated with parental attitudes toward shared reading at all three measurement points (*r* = 0.37–0.39). Children’s level of linguistic skills seemed to be partly dependent on other influencing variables, such as age, gender, intelligence, and the family’s socioeconomic background (mainly small to medium effect size correlations). Whereas the HLE and parental attitudes toward reading to their children were strongly associated with families’ SES (*r* = 0.40–0.58), the correlations with the other control variables were much smaller and mostly not significant.

### Change in Parental Attitudes Toward Shared Reading and the Quality of the Home Literacy Across t1 to t3

In order to investigate whether the necessary conditions for rm ANOVA had been met, Mauchly’s test of sphericity was conducted. The results indicated that the assumption of sphericity had been violated for parental attitudes, with χ^2^(2) = 11.67, *p* < 0.05. Therefore, a Huynh–Feldt correction was applied. For the HLE, Mauchly’s test of sphericity was not violated, with χ^2^(2) = 2.02, *p* < 0.05. We did not find significant effects of time on parental attitudes and the HLE, with *F*(2,130) = 2.11, *p* = 0.13, η^2^ = 0.03 and *F*(2,138) = 0.01, *p* = 0.99, η^2^ = 0.00. Consequently, no significant and meaningful change in the quality of the HLE and parental attitudes toward shared reading was observed between t1 and t3.

### The Association of Parental Attitudes Toward Shared Reading, the Home Literacy Environment, and Children’s Linguistic Competencies

**FIGURE 1:**
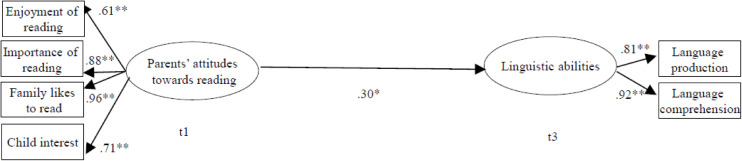
Parental attitudes as a predictor of children’s linguistic abilities. *N* = 133. Standardized beta coefficients with **p < 0.01; *p < 0.05. HLE, home literacy environment. All analyses are controlled for age, sex, intelligence, and parental socioeconomic status (SES) (occupational prestige); χ*^2^*(23) = 37.92, *p* > 0.05, root mean square error of approximation (RMSEA) = 0.07, comparative fit index (CFI) = 0.97, Tucker–Lewis Index (TLI) = 0.94, standardized root mean residual (SRMR) = 0.05.

We used SEM to address the main research question concerning the associations between parental attitudes toward shared reading and children’s linguistic competencies and whether this association may be mediated by the HLE. In a first model, we tested the direct prediction of linguistic competencies by parental attitudes toward shared reading (Figure 1). Latent variables were modeled for parental attitudes and children’s level of linguistic abilities. Here, parental attitudes were modeled using the four items for cognitive attitudes toward reading, whereas the latent linguistic abilities variable included the language comprehension and language production scales of the SETK. In addition, we controlled for children’s age, sex, intelligence, and family’s SES. Parental attitudes toward shared reading at t1 were a significant predictor of children’s linguistic abilities at t3. The proposed model fit the data well, with χ^2^ (23) = 37.92, *p* > 0.05, root mean square error of approximation (RMSEA) = 0.07, comparative fit index (CFI) = 0.97, Tucker-Lewis index (TLI) = 0.94, standardized root mean residual (SRMR) = 0.05.

**FIGURE 2:**
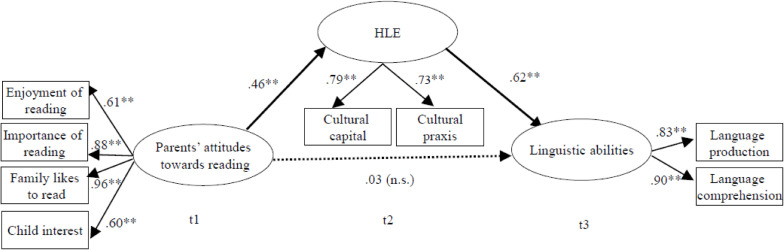
Parental attitudes and their association with children’s linguistic abilities mediated by the home literacy environment. *N* = 133. Standardized beta coefficients with **p < 0.01. HLE, home literacy environment. All analyses are controlled for age, sex, intelligence, and parental socioeconomic status (SES) (occupational prestige); root mean square error of approximation (RMSEA) = 0.04, comparative fit index (CFI) = 0.98, Tucker–Lewis Index (TLI) = 0.97, standardized root mean residual (SRMR) = 0.04.

In the final model (Figure 2), the HLE at t2 was added as a latent variable. The HLE comprised the cultural capital and cultural praxis subscales, and again we controlled for children’s age, sex, intelligence, and family’s SES. When the HLE was added as a mediator to the model, parental attitudes toward shared reading were no longer direct significant predictors of children’s linguistic abilities. Instead, parental attitudes toward shared reading predicted the HLE, which, in turn, predicted children’s linguistic abilities. Consequently, in this full mediation, parents’ attitudes toward shared reading at t1 were significant predictors of children’s linguistic abilities at t3 only indirectly *via* the HLE at t2 with a total standardized indirect effect of 0.28 (*p* < 0.05).

In addition, children’s age was predictive of their linguistic abilities with a standardized beta coefficient of 0.26 (*p* < 0.01), but neither children’s sex, intelligence, nor the family’s SES. However, the family’s SES significantly predicted children’s HLE with a standardized beta coefficient of 0.37, *p* < 0.01, and parent’s attitudes toward reading with a standardized beta coefficient of 0.57, *p* < 0.001. No other control variables were significantly associated with the HLE and parent’s attitudes. The proposed model fit the data very well, with χ*^2^*(27) = 43.09, *p* > 0.05, CFI = 0.98/TLI = 0.97, SRMR = 0.04, RMSEA = 0.04.

## Discussion

The home learning environment that young children experience is a good predictor of early and later literacy and numeracy competencies (Melhuish et al., 2008; Niklas and Schneider, 2017a). Here, aspects such as the onset, frequency, and quality of shared reading which can be summarized as cultural praxis and the number of books at home as an indicator for cultural capital are specific predictors of children’s linguistic and literacy outcomes (McElvany et al., 2009; Niklas et al., 2013; Niklas, 2015). All these aspects are part of a global HLE construct (e.g., Cohrssen et al., 2016; Wirth et al., 2020). However, not much is known about the association of parental attitudes toward shared reading with the HLE, whether these attitudes should be integrated into a broader construct of the HLE or whether they should be treated as an independent variable, and about the association among attitudes, HLE, and children’s linguistic outcomes.

Our findings indicate that whereas the correlations between parental attitudes toward shared reading and the HLE are substantial, there is still reason to differentiate between both constructs (see also Bingham, 2007; Park, 2008). Further support for this differentiation comes from our SEM, as the fit was very good for our final model (Figure 2) that treated parental attitudes and the HLE as separate latent variables. On the other hand, we found large effect size correlations between both constructs, and therefore, it is comprehensible why some studies combined them (e.g., Niklas and Schneider, 2017b). In our view, both operationalizations may be applied in research, depending on the research focus of a study. Here, it is decisive whether parental attitudes toward shared reading are the main research focus and thus should be considered as independent variables or whether they need to be taken into account, but are not in the center of interest, in which case they could be treated as a part of the global HLE.

Our results indicate that parental attitudes toward shared reading might impact on the quality of the HLE parents provide for their children and that, in turn, the HLE positively influences children’s linguistic abilities. Consequently, there was a significant indirect effect of parental attitudes on children’s linguistic outcomes mediated by the HLE. Here, our two SEM models indicate that a full mediation takes place. A significant direct path from parental attitudes toward shared reading was found in Model 1, and this significant association disappeared once the HLE was included as a mediator in the model (Figure 2).

Obviously, the specific parental attitudes toward shared reading that were assessed in our parent survey predicted the literacy behavior of parents in the family context and thus shaped the quality of the HLE (cf. Schwarz and Bohner, 2001, see also Tambyraja et al., 2017). Parents with a more positive attitude toward shared reading also seem to read more often themselves, possess more books, and read more frequently to their child (cf. Niklas, 2015). We further replicated the finding that the early HLE is a very important predictor for young children’s language comprehension and production and that a greater quality in the HLE leads to greater linguistic competencies of children living in such an a HLE (Sénéchal and LeFevre, 2002; Niklas et al., 2013; Niklas and Schneider, 2015; however, see also Puglisi et al., 2017, for a debate of this causal link). Consequently, our results point out that parental attitudes toward shared reading seem to have an indirect impact on child outcomes *via* the literacy interactions that occur in the family context.

Given that our sample consisted of young children who are only about to learn inside-out skills such as phonological awareness and letter knowledge, we focused on language competencies as an indicator for outside-in skills (cf. Whitehurst and Lonigan, 1998). Here, it would be of great interest to test the associations we found for an older sample and inside-out skills. It could be expected that parental attitudes might play an even more important role for such skills as these are closely associated with the formal HLE and thus with aspects such as parental teaching (Sénéchal and LeFevre, 2002). These formal HLE activities were not assessed in our study and would be an interesting target for future studies on parental literacy attitudes.

Another interesting finding is that the parental attitudes toward shared reading are closely associated with the family SES, similarly to the HLE (Niklas and Schneider, 2013, see also Skibbe et al., 2008). SES-related differences in family literacy attitudes and behaviors were also found in elementary school children (Park, 2008; Becker and McElvany, 2018). Our findings indicate that parents with a higher SES (i.e., more prestigious occupations) tend to put more value to shared reading and seem to provide a greater quality HLE for their children. Consequently, it can be assumed that parental attitudes toward shared reading and HLE might act as a mediator between SES and children’s linguistic outcomes (cf. Aikens and Barbarin, 2008; Niklas et al., 2013). In an exploratory SEM analysis, we indeed found significant paths from SES to parental attitudes and the HLE, but the model fit was poorer. The associations between SES, HLE, parental attitudes, and children’s linguistic abilities need to be analyzed in future research.

Given that attitudes and behavior can be successfully changed by interventions (for a recent example in the health context, see Abel Mangueira et al., 2019), parental attitudes may be a very good target for educational interventions. Meta-analyses show that interventions in the HLE may have a positive impact on children’s linguistic and literacy development (e.g., Mol et al., 2008; Sénéchal and Young, 2008), and successful family literacy interventions often include parental education or general information for parents on how to enhance the quality in the HLE (e.g., Niklas and Schneider, 2017b; cf. Saracho, 2017). Such approaches may change attitudes and actual behavior in parents. As attitudes can be changed more easily than, for instance, the socioeconomic background, interventions in the HLE should always consider parental literacy attitudes.

### Limitations

Some limitations mark this study. First, the information about parental attitudes and the HLE was only assessed *via* parental survey and thus may be biased due to social desirability. However, our results are similar to the results of other studies that used parental surveys, and such surveys are often reliable measurement instruments (Bingham, 2007; Skibbe et al., 2008). Other assessments such as children’s book checklists or direct observations in the families (e.g., Sénéchal and LeFevre, 2002) still may have offered a better insight into the association of attitudes, HLE, and child outcomes.

Second, not all children could be assessed at all measurement points, and some parental information was missing. Consequently, the correlational analyses and rm ANOVAs were conducted with a reduced sample. However, the percentage of missing data was similar to those of previous studies on the HLE (e.g., Niklas and Schneider, 2017b). In addition, in our final analyses with SEM, the full information maximum likelihood option in Mplus was applied that takes into account all available information without deleting cases, and thus our results should be reliable.

Third, a small sample of young children participated in our study. Although a power analysis indicated that the sample size was large enough, it would have been preferable to include more children. With a larger sample, it would have been possible to apply a full-forward SEM in which all variables predict all other variables that were assessed at a later time. Further, it would be interesting to assess children with a larger age span to test whether the associations of parental literacy attitudes and the HLE with child linguistic outcomes differ across different age groups. For instance, parents of children who are about to enter school may be more apt to provide a better quality HLE for their children independent of their attitudes toward shared reading. Further, a replication with samples from other countries would be preferable, as the association between our study variables seems to vary across different cultural and economic contexts (Park, 2008).

In addition, our sample was not statistically representative for German children of this age group. However, parental reading habits and the average highest household SES (occupational prestige) in this sample were comparable to other German studies (e.g., Niklas and Schneider, 2017b). Finally, although we included assessments across three measurement points with attitudes assessed at t1, HLE at t2, and linguistic abilities at t3, we still conducted correlational analyses and thus our findings cannot be interpreted as causal associations, although previous studies also point to a causal relation (cf. Hemmerechts et al., 2017).

### Conclusions

The home learning environment is an important predictor for children’s early and later competencies development (Melhuish et al., 2008; Niklas and Schneider, 2017a). Here, shared reading is an important aspect of the HLE and should start early in children’s life and should be part of a regular routine in the family (Niklas et al., 2016a; Wirth et al., 2020). However, little work has focused on the role that parental literacy attitudes play in this context (Bingham, 2007; Skibbe et al., 2008).

In this longitudinal study, we analyzed the associations between parental attitudes toward shared reading and children’s language comprehension and production and found that this association was mediated by the HLE. Further, families with a high SES report more positive attitudes toward shared reading (see also Park, 2008; Becker and McElvany, 2018), and without interventions, such attitudes seem to remain stable across time. Consequently, parental attitudes toward shared reading seem to be an important basis for individual differences in the quality of the HLE and for children’s linguistic competencies and may thus be good targets for family literacy interventions (cf. Saracho, 2017).

## Data Availability Statement

The datasets generated for this study are available on request to the corresponding author.

## Ethics Statement

The studies involving human participants were reviewed and approved by the Ethics Committee, Department of Psychology at the University of Würzburg. Written informed consent to participate in this study was provided by the participants’ legal guardian/next of kin.

## Author Contributions

SE and FN were the PIs of this study. FN wrote the first draft of the manuscript. AW, ND, and SG were responsible for the acquisition of the data. AW and SG conducted the analyses. All authors contributed to the manuscript revision and read and approved the submitted version.

## Conflict of Interest

The authors declare that the research was conducted in the absence of any commercial or financial relationships that could be construed as a potential conflict of interest.
